# Gene Expression Profiles of Melanocytes Over-Expressing miR-5110 in Alpaca

**DOI:** 10.3390/cimb48010093

**Published:** 2026-01-16

**Authors:** Shanshan Yang, Dingxing Jiao, Fengsai Li, Xuqi Wang, Tao Song, Lili Wang, Ping Rui, Zengjun Ma

**Affiliations:** 1 College of Animal Science and Technology, Hebei Normal University of Science and Technology, Qinhuangdao 066000, China; shanshan0321@163.com (S.Y.); linxiao8412@foxmail.com (D.J.); yilvwenrou@126.com (F.L.); 18033571508@163.com (X.W.); songtaoer@126.com (T.S.); iamwanglili100@163.com (L.W.); rp1969@126.com (P.R.); 2 Hebei Key Laboratory of Veterinary Preventive Medicine, College of Animal Science and Technology, Hebei Normal University of Science and Technology, Qinhuangdao 066000, China

**Keywords:** melanogenesis, miR-5110, Kitl, MAPKAPK3, differential expression, gene expression profile

## Abstract

Previous studies have shown that miR-5110 regulates pigmentation by cotargeting melanophilin (MLPH) and WNT family member 1 (WNT1). In order to find the possible molecular mechanism for pigmentation, we examined the mRNA expression profiles in melanocytes of alpaca transfected with miR-5110, inhibitor or negative control (NC) plasmids using high-throughput RNA sequencing. The results showed that a total of 91,976 unigenes were assembled from the reads, among which 13,262 had sequence sizes greater than 2000 nucleotides. According to the KEGG pathway analysis, four pathways related to melanogenesis, the MAPK signaling pathway, Wnt signaling pathway, and cAMP signaling pathway were identified. Compared to the NC, 162 gene were upregulated and 41 genes were downregulated in melanocytes over expressed by miR-5110. The differential expressions of mRNAs Dickkopf 3 (*DKK3*), premelanosome protein (*Pmel*), insulin-like growth factor 1 receptor (*IGF1R*), cyclin-dependent kinase 5 (*CDK5*), endothelin receptor type B (*Ednrb*), kit ligand (*Kitl*), *Myc*, and *S100* were verified using qRT-PCR, which agreed with the results of RNA sequencing. We also verified the differential expressions of mRNAs of some genes in the MAPK signaling pathway using qRT-PCR, which agreed with the results of RNA sequencing. Interestingly, several genes were screened as candidates for the melanogenesis regulated by miR-5110, including Kitl and MAPK-activated protein kinase 3 (*MAPKAPK3*). These findings provide new insights for further molecular studies on the effects of miR-5110 on the melanogenesis and pigmentation.

## 1. Introduction

Melanocytes are specialized cells found in the skin, derived from neural crest cells. They are located at the basal layer of the epidermal hair bulb, eyes, ears, and meninges [1]. During embryonic development, melanoblasts migrate to the basal layer of the epidermis, where they differentiate to mature melanocytes possessing the complete machinery to ensure melanin synthesis and distribution within the skin [2]. Mature melanocytes have an important role in the skin’s innate immunity and determine skin color by producing melanin [3]. Melanin synthesis takes place in intracellular organelles named melanosomes [4]. Melanosomes are specialized, pigment-containing, lysosome-related organelles and can synthesize two types of melanin granules (eumelanin and pheomelanin), and the amount and distribution of the two melanin types determine animal skin and coat color [5]. Additionally, melanin protects against ultraviolet radiation and thus against skin burn and cancer [6]. Melanogenesis is the process by which melanocytes produce melanin in the basal epidermal layers. Although many studies have been conducted on the mechanisms underlying melanogenesis, various aspects of the process remain unclear.

Alpaca (*Vicugna pacos*), a domestic mammal primarily specialized for fiber production [7], exhibits over 22 natural coat colors as a model to study melanogenesis. The coat color formation of mammals includes melanin production in melanocytes and melanins transferring from melanocytes to keratinocytes [8], which is decided and regulated by many genes. Wnt1 is an important gene that plays a role in the menlanogenesis, while MLPH plays a role in melanin transfer in melanocytes [9]. Genes that encode tyrosinase, and the tyrosinase-related proteins (TYRP1 and TYRP2) are commonly involved in the pigmentation disorders caused by many exogenous and endogenous factors, and microphthalmia-associated transcription factor (MITF) plays a fundamental role in the transcriptional regulation of these genes [10].

MicroRNAs (miRNAs) are a class of highly conserved non-coding RNAs, approximately 21–25 nucleotides long, found across various species from plants to mammals [11]. They play a crucial role in gene expression regulation by controlling their target genes at the post-transcriptional level. An increasing number of miRNAs, such as miR-25 [12], miR-137 [13], and lpa-miR-nov-66 [14], have been identified as regulatory molecules that mediate melanogenesis in mammalian melanocytes.

In the previous study, we characterized that miR-5110 regulated rat sarcoma (RAS)-associated binding 27a (RAB27a), myosin 5a (MYO5a), and WNT/β-catenin signaling pathways by cotargeting MLPH and WNT1, which resulted in upregulated eumelanin production through tyrosinase-related protein2 (TYRP2) and downregulated pheomelanin production through TYR (tyrosinase) inalpaca melanocytes [9]. To better understand the regulatory mechanisms linked to the pigmentation or melanogenesis of miR-5110 in the melanocytes of alpaca, we investigated the transcriptome profiles in melanocytes of alpaca transfected with miR-5110, inhibitor, or NC plasmids using high-throughput RNA sequencing. These findings may provide new insights into the regulatory mechanisms of miR-5110 in pigmentation and melanogenesis.

## 2. Materials and Methods

### 2.1. Ethics Statement

All experimental procedures were approved by the Committee on the Ethics of Animal Experiments of Hebei Normal University of Science and Technology, Qinhuangdao, China (approval code: 2025015; approval date: 13 March 2023) and performed according to the committee guidelines.

### 2.2. Cell Culture and Transfection

The miR-5110 expression plasmid was created by inserting an oligonucleotide of the pre-miR-5110 sequence into pcDNA6.2-GW/EmGFPmiR (Invitrogen, Carlsbad, CA, USA). Negative control and inhibitor plasmids were created using a scrambled pre-miR-5110 sequence. Melanocytes, grown to 75–80% confluent, were transfected with pcDNA6.2-miR-5110, inhibitor, or negative control (NC) plasmid using Lipofectamine 2000 (Invitrogen, Carlsbad, CA, USA) following the manufacturer’s protocol.

### 2.3. RNA Sample Preparation, Quantification, and Qualification

Three days after transfection, the melanocytes were collected. Total RNA extraction was performed using TRIzol reagent (Invitrogen, Carlsbad, CA, USA). RNA purity was evaluated with a NanoPhotometer^®^ spectrophotometer (IMPLEN, Westlake Village, CA, USA). RNA concentration was measured using the Qubit^®^ RNA Assay Kit in Qubit^®^ 2.0 Flurometer (Life Technologies, Carlsbad, CA, USA). RNA integrity was assessed with the RNA Nano 6000 Assay Kit of the Agilent Bioanalyzer 2100 system (Agilent Technologies, Santa Clara, CA, USA).

### 2.4. Library Construct for Transcriptome Sequencing

Each transfection group (pcDNA6.2-miR-5110, inhibitor, and NC) included three independent biological replicates, which were processed separately throughout library preparation and sequencing. A total of 3 μg RNA per sample was used for the RNA sample preparations. Sequencing libraries were created using NEB Next^®^ Ultra™ RNA Library Prep Kit for Illumina^®^ (NEB, Ipswich, MA, USA) according to the manufacturer’s recommendations. mRNA was purified from total RNA using poly-T oligo-attached magnetic beads. Fragmentation was carried out using divalent cations at elevated temperature in NEBNext First Strand Synthesis Reaction Buffer (5×). First-strand cDNA was synthesized using random hexamer primer and M-MuLV Reverse Transcriptase (RNase H-). Second-strand cDNA synthesis was subsequently performed using DNA PolymeraseI and RNaseH. Overhangs were converted to blunt ends via exonuclease/polymerase activity. After adenylation of 3′ends of DNA fragments, the NEB Next Adaptor with hairpin loop structure was ligated to prepare for hybridization. To select cDNA fragments of 150–200 bp, the library fragments were purified using the AMPure XP system (Beckman Coulter, Beverly, CA, USA). Next, 3 μL of USER Enzyme (NEB, Ipswich, MA, USA) was added to the size-selected and adaptor-ligated cDNA, and incubated at 37 °C for 15 min, followed by 5 min at 95 °C before PCR. Then, PCR was conducted with Phusion High-Fidelity DNA polymerase, Universal PCR primers, and Index (X) Primer. The PCR products were purified using the AMPure XP system (Beckman Coulter, Inc., Brea, CA, USA), and library quality was assessed with the Agilent Bioanalyzer 2100 system. The prepared libraries were sequenced on an Illumina HiSeq 2000 platform (Illumina, San Diego, CA, USA), and paired-end reads were generated. Data analysis was performed based on the individual replicate sequences, and results represent comparisons across groups rather than pooled samples.

### 2.5. Unigene Assembly and Functional Annotation

Raw reads were cleaned by removing low-quality reads before assembly. Unigene assembly was performed using the Trinity (https://github.com/trinityrnaseq/trinityrnaseq/wiki, accessed on 6 January 2026). The unigene sequences were compared with NR, Swiss-Prot, GO, COG, KOG, eggNOG4.5, and KEGG using BLAST (version 2.16.0). KEGG Orthology results were obtained using KOBAS2.0. The amino acid sequence of unigenes was predicted and compared with Pfam using HMMER (version 3.4) to obtain the annotation information.

### 2.6. Identification of Differentially Expressed Genes and Pathway Analysis

Differential expression analyses of two or three conditions were performed using the DESeq (version 1.48.1) R package. DESeq offers statistical methods to identify differential expression in digital gene expression data using a negative binomial distribution model. The resulting *p*-values were adjusted using Benjamini and Hochberg’s method to control the false discovery rate (FDR). Genes with an adjusted *p*-value <0.05 and an absolute Log2FC (fold change) ≥ 2 were considered differentially expressed. Gene ontology (GO) enrichment analysis of the differentially expressed genes (DEGs) was performed using the topGO (version 2.56) R packages based on the Kolmogorov–Smirnov test. KEGG is a database that helps understand the high-level functions and utilities of the biological systems, such as the cells, the organisms, and the ecosystems, using molecular-level data, particularly large-scale molecular datasets from genome sequencing and other high-throughput experiments. We utilized the KOBAS software to statistically assess gene enrichment in KEGG pathways.

### 2.7. Quantitative Real-Time RCR

Quantitative real-time PCR (qRT-PCR), a conventional quantification method for gene expression, was used to validate the RNA-seq analysis. Based on these results, we optionally identified several coat color genes for validation through quantitative real-time PCR analysis. These genes and their corresponding primers used in the qRT-PCR are listed in Table 1. The qRT-PCR analysis was performed in a volume of 10 µL containing 5 µL 2× SYBR Premix Ex TaqⅡ, 0.2 µL ROX Reference DyeⅡ (Takara Bio Inc., Dalian, China), 0.4 µL of each primer (10µM final concentration), 3.4 µL water, and 1 µL diluted cDNA using the Stratagene Mx3005P system (Agilent Technologies, Santa Clara, CA, USA). Real-time PCR conditions were as follows: an initial denaturation at 95 °C for 10 min, followed by 40 cycles of 95 °C for 5 s, 54 °C for 20 s, and 72 °C for 15 s. Each sample was analyzed in triplicate. mRNA transcript abundance was quantified using the comparative threshold cycle (CT) method [15].

### 2.8. Data Analysis for qRT-PCR

All data were analyzed using GraphPad Prism 5 software (GraphPad Software Inc., San Diego, CA, USA). Statistical analysis was conducted using one-way ANOVA, followed by Tukey’s test. Data were presented as mean ± standard error, with *p* < 0.05 indicating statistical significance (*n* = 3).

## 3. Results

### 3.1. Overview of Unigenes Data from RNA-Seq Analysis

**Figure 1:**
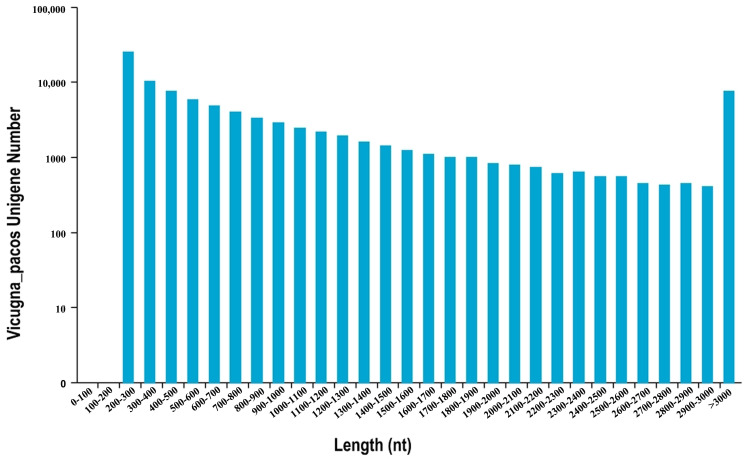
Length distribution and abundance of all unigenes identified in melanocytes.

Following the filtering of raw reads, 41,188,319 clean reads with 53.97% GC percentage and 32,132,258 clean reads with 55.01% GC percentage were obtained from melanocytes transfected with miR-5110 inhibitor and NC plasmids, respectively. These clean reads were assembled into unigenes, obtaining 91,976 unigenes. Of the unigenes, 13,262 had a sequence longer than 2000 nucleotides (Figure 1).

### 3.2. Functional Classification of the Unigenes

**Figure 2:**
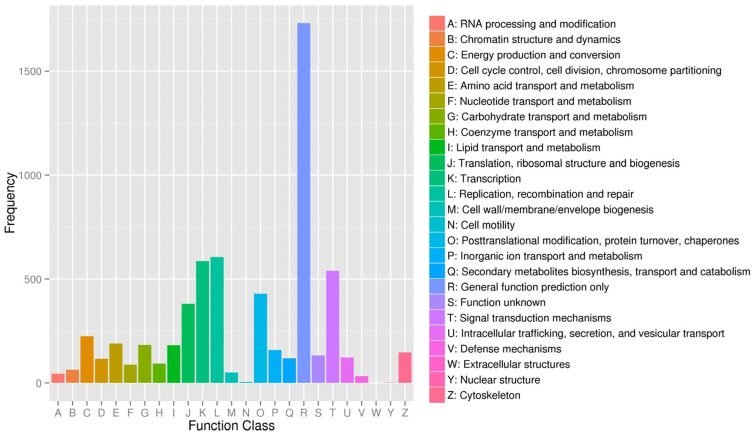
COG functional classification of differentially expressed unigenes. The COG functional classifications of unigenes that were differentially expressed between the melanocytes transfected with miR-5110 and melanocytes transfected with NC are shown.

**Figure 3:**
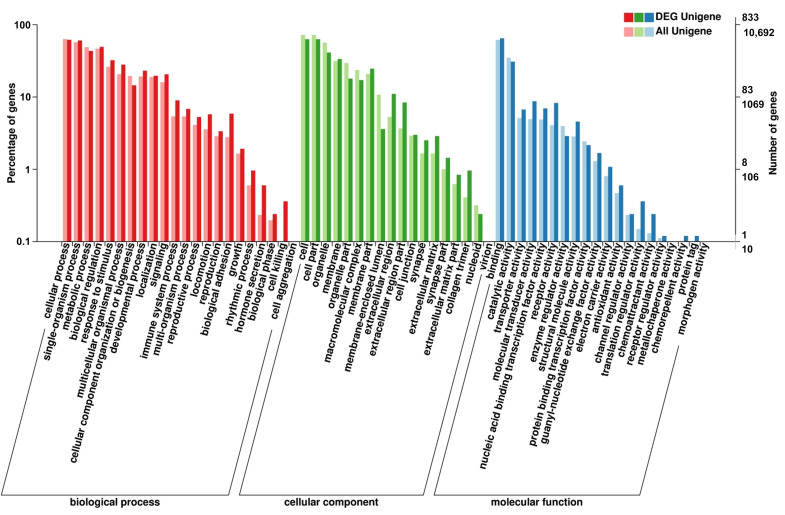
Gene ontology (GO) functional classification of differentially expressed unigenes. The GO functional classifications of unigenes that were differentially expressed between the melanocytes transfected with miR-5110 and melanocytes transfected with NC are shown.

BLAST analysis (E-value < 0.00001) of the unigenes against the protein and nucleotide databases identified 19,988 known genes, with 4527 annotated using COG classification. These genes were categorized into 25 classes based on their putative functions, and the largest group of genes was classified into the predicted general function (27.8%, Figure 2). The known genes were categorized into three classes categories: cellular components, molecular functions, and biological processes (Figure 3).

### 3.3. Divergent Expression Patterns of mRNA

**Figure 4:**
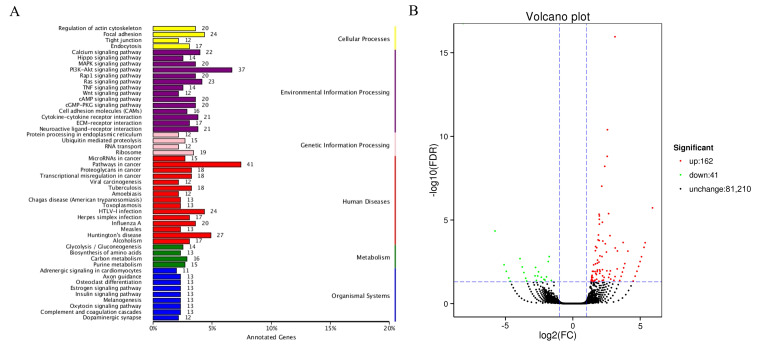
Divergent expression patterns of mRNAs in melanocytes transfected with miR-5110 versus NC. (**A**) KEGG pathway analysis in melanocytes transfected with miR-5110 versus NC. (**B**) Divergent expression patterns of mRNAs in melanocytes transfected with miR-5110 versus NC. The blue dashed line represents the threshold line for differentially expressed genes.

In total, 9789 genes were annotated using KEGG, and 875 genes displayed a specific KEGG pathway annotation, which were grouped into 50 pathways, including those functionally related to coat colors, such as melanogenesis, MAPK signaling pathway [16], Calcium signaling pathway, and Wnt signaling pathway [17] (Figure 4A). Transcriptome sequencing analysis found 203 DEGs in melanocytes transfected with the NC plasmid versus those transfected with the miR-5110 plasmid, including 162 upregulated and 41 down-regulated genes using the Volcano plot (Figure 4B). The top 10 DEGs are shown in Table 2. Among the coat color genes with lower expression in the melanocytes transfected with the miR-5110 plasmid, Kit ligand (Kitl) showed the largest decrease in expression, followed by the lymphoid enhancer factor 1 (Lef-1), melanocyte protein 17 (Pmel), melanophilin (MLPH), and wingless-related MMTV integration site 1 (Wnt1) genes.

### 3.4. Validation of Sequencing Data Using Quantitative Real-Time PCR

**Figure 5:**
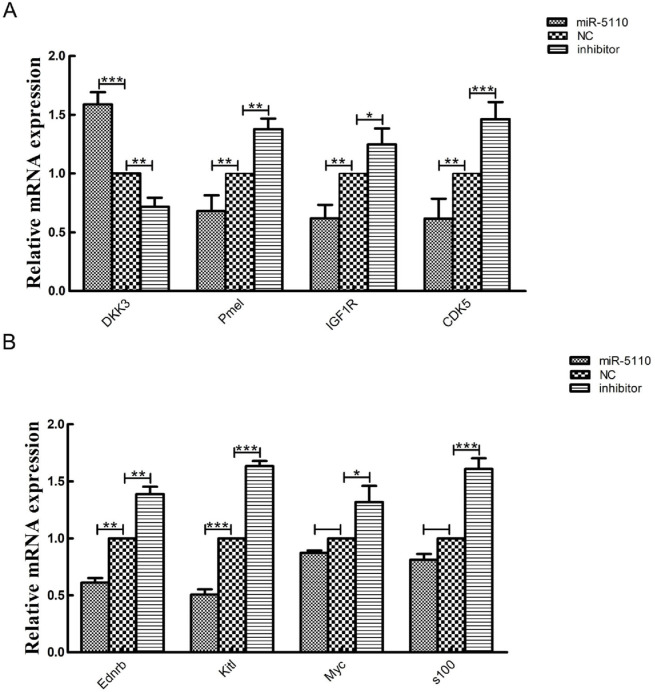
(**A**,**B**) Relative mRNA expression of eight genes involved in melanogenesis in melanocytes transfected with miR-5110 versus NC. Quantitative real-time PCR validation of eight randomly selected genes that were determined to be differentially expressed in melanocytes between those transfected with miR-5110 and the NC plasmid. The abundances of target genes were normalized to the abundance of a β-actin endogenous control. The bars in each panel represent the mean ± standard error (*n* = 3). * *p* < 0.05; ** *p* < 0.01; *** *p* < 0.001.

**Figure 6:**
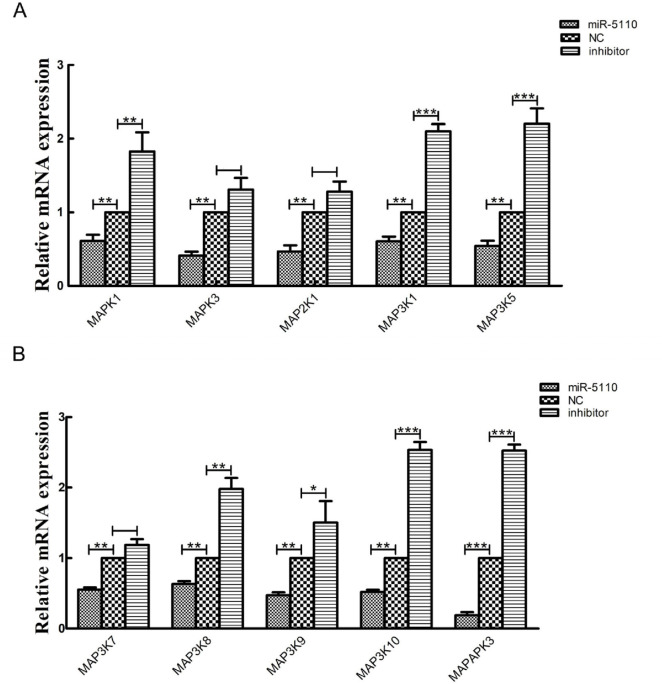
(**A**,**B**) Relative mRNA expression of some genes in the MAPK signaling pathway in melanocytes transfected with miR-5110 versus NC. Quantitative real-time PCR validation of certain genes in the MAPK signaling pathway showed that they were differentially expressed in melanocytes between those transfected with miR-5110 and NC plasmids. The abundances of target genes were normalized to the abundance of a β-actin endogenous control. The bars in each panel represent the mean ± standard error (*n* = 3). * *p* < 0.05; ** *p* < 0.01; *** *p* < 0.001.

To validate the transcriptome sequencing results, we randomly selected eight genes associated with coat color formations and nine genes from the MAPK signaling pathway for validation via quantitative real-time PCR (qRT-PCR). These genes were identified as differentially expressed in the melanocytes transfected with the miR-5110 plasmid compared to those transfected with the NC plasmid, based on transcriptome sequencing analysis. The mRNA expression levels measured by qRT-PCR were in agreement with the transcriptomic data for the selected genes (Figure 5). Among the DEGs, Kitl showed the most significant difference in expression, thereby supporting our sequencing data. Furthermore, based on the RNA-seq analysis of the KEGG pathways, the MAPK signaling pathway was selected for validation via qRT-PCR; statistical analysis of the data revealed that MAPAPK3 showed the greatest difference in expression (Figure 6).

## 4. Discussion

In this study, we identified that miR-5110 transfection led to significant expression changes in several key genes involved in melanogenesis, including DKK3, Pmel, IGF1R, and notably Kit. This suggests that miR-5110 may regulate pigmentation by targeting a network of genes across multiple pathways. For instance, Dickkopf 3 (DKK3), a critical inhibitor of the Wnt/β-catenin signaling pathway [18], was downregulated. Given that DKK3 is known to suppress melanogenesis by downregulating microphthalmia-associated transcription factor (MITF) [19], its decreased expression in our system implies a potential mechanism by which miR-5110 could activate the Wnt pathway to promote pigment production, a hypothesis that warrants further validation.

KEGG pathway analysis indicated that the differentially expressed genes (DEGs) downstream of miR-5110 were significantly enriched in pathways critical for pigmentation, such as the Wnt, MAPK, and cAMP signaling pathways. This places miR-5110 as an upstream regulator of these established melanogenic networks. Specifically, within the Wnt pathway, we observed the downregulation of DKK3, a known inhibitor. This suggests that miR-5110 may potentially alleviate the inhibitory effect on β-catenin, thereby possibly enhancing MITF transcription and subsequent tyrosinase family gene expression [20,21,22]. Similarly, within the MAPK signaling pathway, alterations in genes like MAP3K7 (TAK1) were detected. As MAP3K7 is involved in activating downstream kinases like p38, which in turn phosphorylate CREB and MITF [23,24], the changes induced by miR-5110 could modulate MITF activity at a post-translational level. This multi-level regulation-transcriptional via Wnt and post-translational via MAPK highlights a coordinated mechanism through which miR-5110 might converge on MITF to control melanogenesis.

Among the identified DEGs, Kit ligand (Kitl) showed the largest decrease in expression in the miR-5110 group compared to the NC group. Kit ligand (Kitl), also known as steel factor, stem cell factor, and mast cell growth factor [25], plays a crucial role in melanocyte survival, proliferation, and migration [26], and its receptor c-Kit regulates MITF stability and activity [27,28,29,30]. The dramatic suppression of Kitl suggests a primary mechanism by which miR-5110 influences melanocyte physiology. We hypothesize that miR-5110 may directly target Kitl or an upstream regulator of it. This finding distinguishes miR-5110 from other pigmentation-associated miRNAs. For example, while miR-25-3p is reported to target MITF directly, and miR-145 targets Sox9/10, our data implicate miR-5110 in modulating the Kitl/c-Kit signaling axis, a key pathway for melanocyte development and maintenance. The consequent reduction in Kitl signaling could lead to diminished MITF activity and reduced melanogenic output, providing a plausible explanation for the observed phenotypic effects. Taken together, our results suggest that miR-5110 does not regulate melanogenesis through a single gene but rather orchestrates a concerted effect on a network of pathways. By potentially suppressing inhibitors like DKK3 in the Wnt pathway, modulating kinases in the MAPK cascade, and profoundly downregulating the key mitogen factor Kitl, miR-5110 emerges as a novel multi-faceted regulator that converges on MITF and melanocyte homeostasis to control pigmentation. Future studies validating direct targets of miR-5110 within these pathways will be crucial to solidify this regulatory model. While this study delineates the transcriptomic landscape altered by miR-5110, future work is also needed to confirm these changes at the protein level and to establish direct regulatory relationships through experiments such as Western blot analysis and luciferase reporter assays.

## 5. Conclusions

In summary, this is the first report on the transcriptome analysis of the melanocytes transfected with miR-5110. The present study describes and reveals a set of known DEGs and pathways regulated by miR-5110 in melanocytes that are responsible for the coat colors.

## Figures and Tables

**Table 1:** Primers used for quantitative real-time PCR.

Gene	Primers (5′→3′)	Application
*DKK3*	F: TTACCTCCCAGTTATCACAATG	Real-time PCR
	R: GCACTCGTGGCTCCTTTT	
*Pmel*	F: TTACTGACCAGGTGCCCTTC	Real-time PCR
	R: TCCAAAGTCCCAGGTGTAGG	
*IGF1R*	F: CCTCCACATCCTGCTCATTT	Real-time PCR
	R: GATGACCAGGGCGTAGTTGT	
*CDK5*	F: ATGACGATGATGAGGGAGT	Real-time PCR
	R: AGCTTCTTGTCGCTATGC	
*Ednrb*	F: GTTCCGAAATGGACAGCA	Real-time PCR
	R: CACAAGTCATCAGGGTATAAAA	
*Kitl*	F: CACTGTTGGATAAGCGAGAT	Real-time PCR
	R: CTGCCCTTGTCAGATTGG	
*Myc*	F: GCTGCCAAGAGGGCTAAG	Real-time PCR
	R: TCAGGGATCTGGTCACGAA	
*S100*	F: ATGACGAAGCTGGAGGAG	Real-time PCR
	R: CTTTGGTGTTCTTGAGGG	
*MAPK1*	F: CAAGGGCTACACCAAGTC	Real-time PCR
	R: TCCGAGAATACCCAGAAT	
*MAP2K1*	F: CTACAGCGACGGCGAGAT	Real-time PCR
	R: TGGAGGAGGGATGGGATA	
*MAP3K1*	F: CCCAGACAATAAATACCG	Real-time PCR
	R: GCTACGCCTACTGTGATA	
*MAP3K5*	F: AAGGCACTTACGGGATAG	Real-time PCR
	R: GACCGAAGAAGAGCAGAA	
*MAP3K7*	F: GATGCGGTACTTTCCAGG	Real-time PCR
	R: AAGCGATGTCCATAAACGAG	
*MAP3K8*	F:AGGAGGTGCCGTGGTTGT	Real-time PCR
	R: TTCCCGATTCTTGTGGTC	
*MAP3K9*	F: GGGTGAAGAAACGCAAGG	Real-time PCR
	R: TGTGAATAACAAAGGTCGGATG	
*MAP3K10*	F: GGGCTTTGGCAAGGTCTATC	Real-time PCR
	R: TGTCTGCGAGGTTGTGGTTC	
*MAPKAPK3*	F: GCACGCAGTGACAGATGA	Real-time PCR
	R: CCTGAATCCTGCTGAACAA	
*β-actin*	F: CTAAGGAGAAGGGCCAGTCC	Real-time PCR
	R: CTCAAGTTGGGGGACAAAAA	

F: forward primer; R: reverse primer.

**Table 2:** Top 10 DEGs in melanocytes transfected with miR-5110.

Gene_Name	log2(fc)	Pval	Gene_Name	log2(fc)	Pval
*Kitl*	−7.93	4.1 × 10^−14^	*SCUBE3*	5.28	4.31 × 10^−4^
*Lef-1*	−5.47	7.3 × 10^−10^	*LYNX1*	4.63	2.2 × 10^−2^
*Pmel*	−5.32	1.9 × 10^−11^	*LOC102538411*	4.62	2.2 × 10^−2^
*MLPH*	−4.98	1.05 × 10^−9^	*IGF2BP3*	4.48	4.4 × 10^−2^
*Wnt1*	−4.90	1.5 × 10^−7^	*IQGAP2*	3.77	7.2 × 10^−3^
*MAPK1*	−3.75	6.4 × 10^−3^	*ARHGAP44*	3.68	1.2 × 10^−2^
*MAPKAPK3*	−3.42	4.4 × 10^−2^	*TGFβ1*	3.29	8.3 × 10^−3^
*CRYM*	−2.77	6.8 × 10^−3^	*LOC140698870*	3.13	2.4 × 10^−2^
*EDN2*	−1.757	1.5 × 10^−3^	*TGM6*	3.12	1.1 × 10^−16^
*P2RX7*	−1.59	4.1 × 10^−2^	*C9H16orf87*	2.81	3.4 × 10^−2^

## Data Availability

The RNA-Seq data presented in this study are available in BioProject database at https://submit.ncbi.nlm.nih.gov/subs/sra/SUB13842719/overview (accessed on 6 January 2026) under the accession numbers PRJNA1018659. The data used and analyzed during this study are available from the corresponding author upon reasonable request.
